# Long-term Benefits of Risk Factor Reduction in Takotsubo Cardiomyopathy.—A Comment on Khalighi *et al*. Entitled “Takotsubo Cardiomyopathy: A Long Term Follow-up Shows Benefit with Risk Factor Reduction”, *J. Cardiovasc. Dev. Dis*., 2015, *2*, 273–281.

**DOI:** 10.3390/jcdd3010003

**Published:** 2016-01-25

**Authors:** Shiva P. Ponamgi, Abhishek Deshmukh

**Affiliations:** 1 Division of Hospital Internal Medicine, Mayo Clinic Health System, Austin, MN 55912, USA; Ponamgi.Shiva@mayo.edu; 2 Division of Cardiovascular Diseases, Mayo Clinic, 200 First Street SW, Rochester, MN 55905, USA

**Keywords:** Takotsubo cardiomyopathy, risk factors, long term

Takotsubo cardiomyopathy (TC) or stress-induced cardiomyopathy is also popularly referred to as “broken heart syndrome” or “apical ballooning syndrome”. It resembles acute myocardial infarction (AMI) in its clinical and electrocardiographic presentation but with no evidence of significant epicardial coronary artery disease [1,2]. TC is most often reported in post-menopausal women with recent sudden, unexpected, emotional or physical stress [3]. The name “Takotsubo cardiomyopathy” comes from the striking similarity between a Japanese fishing pot (“tako-tsubo”) used to catch octopi and the apical ballooning of the left ventricle seen in patients with this condition [4]. Despite many well-documented reports of TC that have been published, the actual pathophysiology of TC and any modifiable risk factors associated with it are yet to be unequivocally established.

In this current journal issue, Khalighi *et al.* report improved long-term outcomes in 12 TC patients (mean follow up 8.3 ± 3.6 years) when prescribed therapeutic lifestyle changes (TLC) and guideline-directed medical therapy (GDMT) for aggressive risk factor reduction [5]. The authors report that the use of cardio-selective beta blockers and calcium channel blockers along with ACE inhibitors may prevent coronary vasospasm in TC patients caused by catecholamine-induced overdrive, suggesting it to be one of the main underlying causes for developing TC. The multivessel epicardial coronary artery spasm causing regional stunning of the myocardium as reported by Kurisu *et al.* [4] would still not explain the severe apical ventricular dysfunction with no significant elevation of cardiac enzymes as seen in TC patients. Moreover, as eluded to earlier by Akashi *et al.*, most reported cases of TC have no clear documented evidence of spontaneous or inducible coronary arterial spasm, making this a less likely cause for developing TC and suggesting an alternate pathophysiology [6]. 

Toxic levels of local catecholamines may be the main culprit behind the cardiotoxicity and the resulting severe left ventricular (LV) dysfunction seen in TC patients. The use of beta blockers in the long term may, in fact, play a protective role by shielding the heart from further such adrenergic surges and thereby allowing recovery. The ACE inhibitors such as in cardiomyopathy from any other etiology could decrease the afterload on the heart, leading to subsequent improvement in the LV function. The preponderance of adrenoceptors at the LV apex as compared to the base may be responsible for the selective apical cardiac toxicity seen in TC patients resulting in the classic apical ballooning pattern described previously [7]. Increased catecholamine levels can stimulate platelet activation, causing transient thrombosis or microvascular dysfunction and, therefore, antiplatelet therapy could in theory decrease the risk of future cardiac events as described by the authors. More recently, the use of aspirin or dual platelet therapy (aspirin and clopidogrel) at the time of hospitalization was shown to be independently associated with lower incidence of in-hospital major adverse cardiac events (MACE), further corroborating these findings [8]. 

In summary, the acute, severe and unexpected emotional/physical stressor that triggers the initial catecholamine surge is probably the most important risk factor in developing TC. The relatively low probability of recurrence of such an event again itself might afford significant protection from developing future adverse cardiac events in patients with complete recovery. The retrospective nature of the study, the small sample size along with the lack of a control group and the presence of survivor bias remain the main limitations of this study and have been appropriately acknowledged by the authors.
